# Shape-memory collagen scaffold combined with hyaluronic acid for repairing intervertebral disc

**DOI:** 10.1186/s40824-023-00368-9

**Published:** 2023-03-29

**Authors:** Young Won Koo, Chang Su Lim, Anjani Darai, JiUn Lee, Wonjin Kim, Inbo Han, Geun Hyung Kim

**Affiliations:** 1 Department of Precision Medicine, Sungkyunkwan University School of Medicine, Suwon, 16419 Republic of Korea; 2 Department of Neurosurgery, CHA University School of Medicine, CHA Bundang Medical Center, Seongnam-Si, Gyeonggi-Do 13496 Republic of Korea; 3 Department of Biophysics, Institute of Quantum Biophysics , Sungkyunkwan University, Suwon, 16419 Republic of Korea

**Keywords:** Collagen-cryogel, Shape-memory material, Hyaluronic acid, Intervertebral disc regeneration

## Abstract

**Background:**

Intervertebral disc degeneration (IVDD) is a common cause of chronic low back pain (LBP) and a socioeconomic burden worldwide. Conservative therapies and surgical treatments provide only symptomatic pain relief without promoting intervertebral disc (IVD) regeneration. Therefore, the clinical demand for disc regenerative therapies for disc repair is high.

**Methods:**

In this study, we used a rat tail nucleotomy model to develop mechanically stable collagen-cryogel and fibrillated collagen with shape-memory for use in minimally invasive surgery for effective treatment of IVDD. The collagen was loaded with hyaluronic acid (HA) into a rat tail nucleotomy model.

**Results:**

The shape-memory collagen structures exhibited outstanding chondrogenic activities, having completely similar physical properties to those of a typical shape-memory alginate construct in terms of water absorption, compressive properties, and shape-memorability behavior. The treatment of rat tail nucleotomy model with shape-memory collagen-cryogel/HA alleviated mechanical allodynia, maintained a higher concentration of water content, and preserved the disc structure by restoring the matrix proteins.

**Conclusion:**

According to these results, the collagen-based structure could effectively repair and maintain the IVD matrix better than the controls, including HA only and shape-memory alginate with HA.

## Background

Chronic low back pain (LBP) is strongly associated with intervertebral disc (IVD) degeneration (IVDD) and is the prime cause of disability worldwide; about 60–80% of adults experience LBP [1]. The etiology and pathogenesis of chronic LBP are complex and multifaceted. The various factors responsible for IVDD such as aging, cell loss, extracellular matrix (ECM) anabolism, and catabolic imbalance result in the breakdown of the disc tissue microenvironment, which can lead to more severe conditions such as disc herniation, disc bulging, and spinal stenosis [2–6].

In general, hydrogels have been widely applied in various tissue engineering applications, including drug delivery systems and self-fitting shape-memory constructs, for minimally invasive surgery [7–12]. Owing to the unique properties of hydrogels, such as high-water content, biocompatibility, tunability, and biodegradability, the development of hydrogels with a mechanism similar to that of disc tissue has been investigated for the treatment of IVDD [13]. Collagen is a primary component of the IVD cellular matrix and can be used in various forms, such as thin films, 3D porous structures, nanofibrous shapes, nanoparticles, and hydrogel [14–19]. In particular, the use of collagen as an injectable hydrogel is a promising choice for successfully regenerating annulus fibrosus (AF) and nucleus pulpous (NP) tissues involved in IVDD [14]. For instance, injectable hydrogels using fibrin and type I collagen, which were crosslinked with genipin, were implanted in the AF region and they showed significant in vitro cellular activities and sealing effect on the AF defect in an in vivo model that used rat tail [18, 19].

Hyaluronic acid (HA) is a distinctive glycosaminoglycan (GAG) and an abundant polymeric component of the ECM that is widely used to treat osteoarthritis and repair IVDD through tissue engineering [20–22]. HA is used as an anti-inflammatory, analgesic, anti-apoptotic, and matrix modulatory biomaterial as well as a delivery vehicle for mesenchymal stem cells (MSCs) in discs [23, 24]. Combining HA with various biocompatible and biodegradable biomaterials such as collagen and fibrin can reduce inflammation and pain in the IVD [25, 26]. Many small and large animal models have been used to test the efficacy of HA and HA-based biomaterials in a preclinical trial of IVDD [23]. In a clinical trial, the implantation of HA in combination with adipose-derived MSCs reduced back pain and enhanced the function and quality of life of patients [27]. In addition, HA-based materials have been used to treat the NP and AF regions of IVD tissue because they act as a reservoir for growth factors, therapeutic drugs, and vehicles for cell delivery [24, 28, 29]. Several studies have reported the ability of HA to alleviate pain and inflammation due to IVDD, enhance the synthesis of ECM proteins, and restore matrix proteoglycans (PGs) in IVD [23].

However, injection of hydrogels is limited by several challenges such as toxicity, gelation time, gel formation, poor mechanical properties, and the ability to protect the biomolecules (cells/drugs) in the biological environment. In addition, liquid precursor solutions may leak into the surrounding tissues, which may alter gel properties and limit hydrogel formation [30, 31]. On the other hand, solidified/crosslinked hydrogels with hard stiffness may experience structure failure during the injection process due to poor adaptability to the uneven defective regions. Therefore, producing an injectable hydrogel with proper shape-memory properties and scaffold flexibility is of great importance.

Fabrication methods of shape-memory hydrogels for IVDD can vary according to the material properties of the hydrogels, such as HA, alginate, elastin, collagen, etc. [32–39]. In particular, Guillaume et al. reported an alginate-based hydrogel with shape-memory and high porosity obtained using phase separation and freeze-drying method [32]. Since alginate has exhibited acceptable mechanical properties, the alginate shape-memory hydrogel could be applicable to the treatment of IVDD. However, alginate hydrogels showed poor cellular activities due to the lack of cell-binding sites available for cell adhesion and proliferation. To overcome these issues, several researchers have been performed to identify a hybrid material composed of alginate with shape-memory properties, good mechanical strength, and coated collagen to induce biological activities [34]. One such study demonstrated the usefulness of the physical properties of injectable structures with shape-memory on in vitro cellular activities, including cell migration of AF and scaffold colonization. Injectable alginate/collagen scaffolds with shape-memory showed promising results in in vitro and ex vivo experiments; however, the ability of the collagen to completely coat the shape-memory alginate structure and a clear evaluation in an in vivo animal model were not performed.

Generally, collagen scaffolds have not been used in IVD regeneration because of their relatively poor mechanical properties and complex fabricating process, despite of the fact that they have superior biological properties compared with that of conventional alginate-shape-memory constructs. However, in our previous study, collagen-based scaffold with shape-memory behavior could be fabricated into various micro/nanostructures using appropriate processing conditions (pH, temperature, fibrillation, crosslinking, etc.) [33]. Especially, fibrillated collagen shape-memory hydrogel with nano/micropores has shown significant enhancement (about 6.3 times) in mechanical properties compared to simply freeze-dried collagen shape-memory hydrogel.

Therefore, in the present study, we prepared three different types of hydrogels with shape-memory: freeze-dried alginate-cryogel and two types of collagen shape-memory structures (freeze-dried collagen-cryogel and fibrillated (or fibrous) collagen) for comparison of their effectiveness in the treatment of IVDD. Although robust shape-memory properties and high-water absorption was observed in all three structures, a more active shape-memory property was assessed in the alginate and collagen-cryogels than in fibrillated collagen. Furthermore, various in vitro cellular activities of alginate-cryogel, collagen-cryogel, and fibrous collagen were examined in human adipose-derived stem cells (hASCs), which have been successfully and widely used in the regeneration of IVD tissues [24, 40, 41]. In association with the previous studies, the collagen shape-memory structures have shown higher cellular responses than alginate-cryogel. Finally, the shape-memory structures supplemented with HA were studied in a rat tail nucleotomy model with focus on the feasibility for repairing NP in the disc.

## Methods

### Materials

Sodium alginate powder combined with a mixture of mannuronic acid and guluronic acid (M/G ratio of 0.42; LF10/60; FMC Biopolymer, Drammen, Norway) was used to prepare a 4-wt% solution in triple-distilled water (3DW). Calcium chloride (CaCl_2_) powder was purchased from Sigma-Aldrich (St. Louis, MO, USA) and prepared in a 2% solution using 3DW for alginate crosslinking. Type I collagen solution (4 wt%) dissolved in 0.03 N hydrochloric acid (Matrix-PSP, purity: > 98%, pH 4.0) from porcine skin was purchased from MSBio (Gimhae, South Korea). The acidic collagen solution was lyophilized, re-dissolved at a higher concentration (8 wt%), and mixed with 0.25 M NaHCO_3_ at 1:1 ratio to prepare 4 wt% neutralized collagen solution (pH 7.8). 1-ethyl-3-(3-dimethylaminopropyl) carbodiimide hydrochloride (EDC) and N-hydroxy-succinimide (NHS) were purchased from Sigma-Aldrich to prepare EDC (100 mM):NHS (25 mM) solution dissolved in 75% EtOH for collagen crosslinking. Hyaluronic acid (1200–1900 kDa; Novamatrix, Drammen, Norway) was prepared in 1% solution in PBS and sterile-filtered before use for in vivo experiments.

### Fabrication and characterization of shape-memory structures

Cylindrical polylactic acid (PLA) molds (diameter: 5 mm and depth: 5 mm) were prepared using a 3D printer (Single Plus – 320C; CUBICON, South Korea) for fabrication of shape-memory structures (SMSs). For alginate SMS (A-SMS) and collagen-cryogel (CG) preparation, the sodium alginate (4 wt%) and acidic collagen (4 wt%, pH 4.0) solutions were placed in the 3D-printed molds and froze at -20 ℃. The frozen hydrogels were then lyophilized for 24 h. Then, they were crosslinked at 37 ℃ using CaCl_2_ solution for alginate for 1 h and EDC/NHS solution for collagen for 2 h, respectively. For fibrous collagen-gel (FCG) preparation, the neutralized collagen (4 wt%, pH 7.8) solution was placed in the 3D-printed molds and incubated in phosphate-buffered saline (PBS) at 37 ℃ for 24 h to induce collagen fibrillation, followed by treatment with EDC/NHS at 37 ℃ for 2 h. All the crosslinked scaffolds were washed with 3DW.

The surface and cross-sectional morphology of the scaffolds were observed using optical and scanning electron microscopy (SEM, SNE-3000 M, SEC Inc., South Korea).

The pore size distribution of the scaffolds was evaluated using a porosimeter (Autopore V 9620; Micromeritics Inc., United States). The pore diameter and cumulative volume were used to calculate the normalized pore volume density (normalized density) using the following equation:

$$\Delta {V}_{i}/(\Delta {r}_{i}\times {V}_{max})$$

where $\Delta {V}_{i}$, $\Delta {r}_{i}$, and ${V}_{max}$ are the incremental pore volume, pore-radius interval, and maximum cumulative pore volume, respectively.

To measure the water contact angles, optical images of the droplets of rhodamine-containing water (20 μL) on the scaffolds were obtained at room temperature (*n* = 3). The contact angles of the droplets were measured using FiJi software (National Institutes of Health, USA).

To examine the water uptake ability, cuboid (width: 1 mm, height: 7 mm, and thickness: 0.1 mm) samples of A-SMS, CG, and FCG were prepared using 3D-printed molds using the same processes as previously described for alginate and collagen. The samples were fixed using tweezers, and their lower surfaces (1 mm) were immersed in rhodamine dye solution (Sigma-Aldrich). Optical images were captured 5 s after immersion of the lower parts (0–1 mm height) of the cuboid samples into the rhodamine dye solution (Sigma-Aldrich).

The water absorption of the scaffolds was determined by weighing the samples before and after soaking them in distilled water for 2 h. The percentage of increase in the water absorption (%) was calculated using the following equation:

$$\frac{{W}_{2h}-{W}_{i}}{{W}_{i}}\times 100 \left(\%\right)$$

where W_i_ and W_2h_ are the weights of the sample before and after 2 h of soaking in water (*n* = 5).

The specific chemical structures of alginate or collagen SMSs were determined using a Fourier-Transform Infrared (FT-IR) spectrometer (model 6700; Nicolet, USA). The mean of 30 scans at 500–4000 cm^−1^ at a resolution of 8 cm^−1^ was used to represent the FT-IR spectra.

### Mechanical properties and shape recovery ability

Cylindrical samples (diameter: 5 mm; height: 2 mm; *n* = 3) were subjected to compression tests using a universal testing machine (UTM; Top-tech) at a pressing speed rate of 0.1 mm s^−1^. The compressive moduli were determined in the linear region of the stress–strain curve. Cyclic compression tests were performed using the UTM at a constant pressing/relaxing speed (0.1 mm s^−1^).

### In vitro experiments

Human adipose-derived stem cells (hASCs, PT-5006; Lonza, Basel, Switzerland) were cultivated in Dulbecco’s modified Eagle’s medium (DMEM)-low glucose (Hyclone, United States) containing 10% fetal bovine serum (FBS; Biowest, France) and 1% penicillin/streptomycin (PS; Thermo Fisher Scientific, USA) at 37 ℃ in a 5% CO_2_ environment. For in vitro tests, hASCs were harvested and seeded on alginate-cryogel, CG, and FCG scaffolds at 1 $\times$ 10^3^ cells per scaffold. To induce chondrogenic differentiation after 5 days of culture in the growth medium, the hASCs-seeded scaffolds were incubated in a differentiation medium consisting of DMEM-low glucose, 10% FBS, 1% PS, 0.05 mM ascorbic acid (Sigma-Aldrich), 0.1 mM dexamethasone (Sigma-Aldrich), transforming growth factor-β1 (TGF-β1; 10 ng mL^−1^; Sigma-Aldrich), and 1% ITS + (Sigma-Aldrich) at 37 ℃ in a 5% CO_2_ environment. The medium was changed every 2–3 days.

Protein absorption of the SMSs was measured using a bicinchoninic acid (BCA) protein assay kit (Pierce Kit; Thermo Scientific, Waltham, MA, USA). Cylindrical samples (diameter: 5 mm and height: 2 mm) were incubated in culture media containing 10% FBS at 37 °C for 1, 2, 4, 6, or 12 h. At each time point, the specimens were gently washed with PBS and lysed with 0.1% Triton X-100 (0.5 mL) overnight. An aliquot of the lysate (25 μL) was added to 200 μL BCA working reagent in a 96-well plate and incubated for 30 min at 37 °C. Absorbance at 562 nm was determined using a microplate reader (EL800; Bio-Tek Instruments, USA). Specimens incubated in serum-free medium were used as blank controls. Protein adsorption was calculated as the mean ± SD (*n* = 4).

Cell seeding efficiency was analyzed using the MTT assay (Cell Proliferation Kit I, Boehringer Mannheim, Germany) following the manufacturer’s protocol after a day of cell cultivation. Briefly, Cells seeded on the scaffolds were incubated in an MTT reagent (0.5 mg mL^−1^) for 4 h at 37 ℃. The absorbance was measured at 570 nm using a microplate reader (*n* = 5). The cell-seeding efficiency was evaluated as the ratio of the number of cells attached to the scaffold to the total number of seeded cells.

The hASCs seeded on the samples were treated with 0.15 mM calcein AM and 2 mM ethidium homodimer-1 at 37 ℃ for 60 min to stain the live (green) and dead (red) cells, respectively, after 1 day from the cell seeding. The stained samples were observed under a confocal microscope (LSM 700; Carl Zeiss, Germany). Cell viability was calculated by calculating the ratio of the live cells to the total number of cells.

The morphology of the hASCs was observed by staining the cell nuclei, F-actin, and vinculin. Before staining, the cultured cell samples were prepared by treating them with 3.7% formaldehyde (Sigma-Aldrich) for 1 h for fixation and 0.1% Triton X-100 (Sigma-Aldrich) containing 2% bovine serum albumin (BSA; Sigma-Aldrich) for 2 h at 37 ℃ for permeabilization and blocking of non-specific antibody binding. After the samples were treated with an anti-vinculin (5 μg/mL, Abcam, USA) primary antibody at 4 ℃ overnight, the cell nuclei, F-actin, and vinculin were stained for 1 h with diamidino-2-phenylinodole (DAPI; 1:100 dilution in PBS; Invitrogen, USA), Alexa Fluor 594 phalloidin (1:100 dilution in PBS; Invitrogen), and Alexa Fluor 488-conjugated secondary antibodies (1:50 in PBS; Invitrogen), respectively. The stained samples were observed under a confocal microscope (LSM 700). The Fiji software was used to evaluate the fluorescent images.

### In vitro glycosaminoglycan (GAG) production analysis

Alcian blue staining and quantification of the GAG content were performed to evaluate the chondrogenesis of hASCs. The cultured hASC samples were fixed with 10% neutral buffered formalin (NBF) for 30 min and treated with Alcian blue 8GX (0.05 wt/v%; Sigma-Aldrich) dissolved in a sodium acetate buffer (50 mM, pH 5.8; Sigma-Aldrich) supplemented with MgCl_2_ (50 mM; Sigma-Aldrich) for 3 h at room temperature. The stained samples were washed twice with 3DW and the surfaces and cross-sections of the samples were observed using an optical microscope (*n* = 6). For the quantification of GAG content in the cultured cell-samples, the specimens were chemically solubilized using a papain extraction solution and assayed using the Blycan™ Sulfated Glycosaminoglycan Assay Kit (Biocolor Life Sciences Assays, UK) according to the manufacturer’s protocol. Briefly, the samples were treated at 65 ℃ for 3 h with a papain extraction reagent (Sigma-Aldrich) dissolved in 0.2 M sodium phosphate buffer (pH 6.4) and supplemented with sodium acetate (8 mg mL^−1^), ethylenediaminetetraacetic acid (EDTA) disodium salt (0.4 mg mL^−1^; Sigma-Aldrich), and cysteine HCl (0.8 mg mL^−1^; Sigma-Aldrich). The GAGs were stained using 1,9-dimethyl-methylene blue dye reagent for 30 min after discarding the supernatant. The stained GAGs were then dissolved using a dissociation reagent to measure the optical density at 656 nm using a microplate reader. The quantified GAGs were evaluated using predetermined standards. All values are expressed as the mean ± SD (*n* = 6).

### In vivo experimental design

For in vivo experiments, 40 Sprague Dawley rats were randomly divided into five groups: damaged only (G1), (damaged and) injected with hyaluronic acid (HA) only (G2), A-SMS with HA (G3), CG with HA (G4), and FCG with HA (G5) (*n* = 8 per group). Normal discs proximal to the treated discs in each tail were used as the controls. We evaluated pain behaviour 2 days before surgery and up to 6 weeks after surgery using von Frey filaments. Six weeks after surgery, the animals were euthanized, and the coccygeal discs were collected from each rat for radiological and histological analyses.

### Animal model of nucleotomy for disc regeneration study

In this experiment, we induced disc damage in rats using a nucleotomy model, a typical IVDD model that NP was removed through incised AF [42]. Before surgery, eight-week-old female Sprague Dawley rats (220–240 g, Orient Bio Inc.) were put under general anesthesia by intraperitoneally injecting a mixture of Zoletil® (50 mg/kg Virbac Laboratories, France) and Rompun® (10 mg/kg, Bayer, Korea). The tail and pelvic area were sterilized with 70% alcohol and povidone-iodine. A 1 cm incision was made longitudinally along the coccygeal disc, exposing the lateral portion of the tail. Then, a no. 11 scalpel blade (1.5 mm) was inserted into the coccygeal disc (Co5-6), the AF of the disc was cut longitudinally, and nucleotomy was performed by aspiration of the NP with a 22-gauge needle on a 5 mL syringe. The implantation of the regenerative scaffolds was then performed, followed by the intradiscal injection of HA (1 w/v%, 15 μL), using a 25-gauge catheter, at the Co5-6 nucleotomy site in all 40 rats. To avoid the risk of scaffold from stuck in the needle while injecting together with HA, we first implanted the regenerative scaffolds with the help of tweezer and then immediately injected the HA solution to be absorbed into the scaffold for shape recovery. Finally, the skin at the surgical site was sutured and disinfected with povidone-iodine. All animals were kept on heating pads to prevent hypothermia, and 0.9% normal saline (5 mL) was injected subcutaneously. All animals were injected with an antibiotic (Cefazolin, CKD Pharmaceuticals, Seoul, South Korea) and an analgesic (Ketoprofen, SCD Pharm. Co., Ltd., Seoul, South Korea) prophylactically for 3 days after surgery. All animals were kept in an environment with a controlled temperature of 22 ± 1 °C, a relative humidity of 50% ± 1%, and a 12/12 h light/dark cycle. The animal study procedures were approved by the Institutional Animal Care and Use Committee (IACUC) of CHA Bundang Medical Center (IACUC210046), and animal surgeries were performed according to the guidelines.

### Mechanical allodynia

Von Frey test was performed 2 days before surgery (pre). After surgery (post) the test was performed on the days 2, 7, 14, 21, 28, 35, and 42 to check the mechanical allodynia and pain behavior in rats. The rats were individually placed in six-compartmented enclosures with wire mesh floors and lids with air holes for a 20-min habituation period to minimize exploratory activity. The test was administered by using a 2 g filament to apply a force to the ventral surface of the tail-base for a maximum of 6 s, which was sufficient to buckle it slightly. A positive response occurred when the rats responded to the filament by flinching, licking, withdrawing, or shaking the base of the tail immediately or within 6 s. A negative response was recorded if the animal did not show any response, and the test was repeated with the next heavier filament. If a positive response occurred, the same of lower-weight filament (grams) that elicited the response was used for further tests, and a 50% withdrawal threshold was calculated. This test was performed five times for each rat [43]. The von Frey analysis was performed by two observers who were blinded to the animal groups and treatment materials.

### Magnetic Resonance Imaging (MRI)

Magnetic resonance imaging (MRI) was performed 6 weeks after the treatment, and a T2-weighted 9.4 T MRI spectrometer (Bruker BioSpec, USA) was used to study the changes in the disc structure and signal intensity of the disc. Coronal T2-weighted imaging was set as follows: time to repetition (TR), 5000 ms; time to echo (TE), 50 ms; 600 horizontal × 200 vertical matrix; field of view, 60 horizontal × 20 vertical; and 0.8 mm slices. Axial T2-weighted imaging was set as follows: TR, 5000 ms; TE, 30 ms; 150 horizontal × 150 vertical matrix; field of view, 15 horizontal × 15 vertical; and 0.5 mm slices with 0 mm spacing between each slice. The signal intensity of the T2 MRI images was used to evaluate the degree of coccygeal disc degeneration and water content. The area with bright signal intensity in the coronal region of the T2 image was considered the outline of the NP, and the region of interest (ROI) was measured using Image J software (National Institutes of Health, Bethesda, MD, USA) [44]. The signal intensity and MRI index (the product of the NP area and average signal intensity) were evaluated to measure NP degenerative changes [44–46]. Two independent observers who were blinded to the information regarding the treatment materials measured the MRI index.

### Histological analysis

Six weeks after injury and treatment, the rats were sacrificed, and the discs were collected for histological analysis to evaluate tissue structure and morphology. First, safranin-O staining was performed. The discs with adjacent vertebral bodies were fixed in 10% neutral buffered formalin for 1 week and decalcified in RapidCal Immuno (BBC Biochemical, Mount Vernon, WA, USA) for 2 weeks. The tissues were then processed for paraffin embedding and sectioned into coronal Sects. (10 µm) using a microtome (Leica). The sections were dewaxed, rehydrated, and stained with Safranin-O (Sigma, USA) to determine the quantity and distribution range of PGs. The stained sections were then mounted using a mounting media and scanned with an OLYMPUS C-mount camera adapter (U-TVO.63XC, Tokyo, Japan).

In a similar manner, hematoxylin and eosin (H&E) staining was performed. The sections were stained with Mayer’s H&E stain for the evaluation of tissue structure and PG distribution. For the analysis, the disc NP area and cell number were measured using ImageJ software, and only one disc from each rat was analyzed.

Furthermore, the histological grading criteria of the stained tissue sections were scored according to the five histological classification categories. The scores were recorded based on NP morphology (score 0–2), NP cellularity (score 0–2), AF morphology (score 0–2), endplate morphology (score 0–2) and boundary between AF and NP (score 0–2) [47]. Therefore, based on these categories, the score recorded for a normal disc is considered as 0, a mild degenerated disc as 1, and a severe degenerated disc as 2. A total score of 0 represents a normal or healthy disc and a maximum score of 16 represents an injured and severely degenerated disc [47]. All histological specimens were subjectively assessed for tissue morphology, structure, and histological scores by pathologists who were blinded to specimen information.

### Immunofluorescence

After being euthanized at 6 weeks following treatment, the coccygeal discs were collected from the rats and fixed overnight in a 4% paraformaldehyde (PFA) solution and decalcified in a decalcification solution (RapidCal Immuno, BBC Biochemical, Mount Vernon, WA, USA) for 2 weeks. Immunofluorescence analysis was performed for Tie-2 (disc NP progenitor marker), brachyury (disc NP marker), aggrecan (disc matrix component), collagen type II (disc NP matrix component), collagen type I (disc AF matrix component), calcitonin-gene related peptide (CGRP; pain marker), matrix metalloproteinase-13 (MMP-13, catabolic enzymes), tumor necrosis factor alpha (TNF-α), and interleukin-1β (IL-1β) (pro-inflammatory cytokines). The discs were embedded in paraffin wax and sectioned longitudinally using a microtome (Leica) into 5–10 µm-thick sections. Thereafter, the sections were dewaxed, rehydrated, and stained with primary antibodies against Tie-2 (1:200; R&D Systems, USA), brachyury (1:200; Santa Cruz, USA), aggrecan (1:1000; Abcam, UK), collagen type II (1:200; Abcam, UK), collagen type I (1:200; Abcam, UK), CGRP (1:200; Abcam, UK), MMP-13 (1:200; Abcam, UK), TNF-α (1:200; Abcam, UK), and IL-1β (1:200; Novus Biologicals, USA) overnight at 4 °C. The sections were then washed with PBS and Tween 20 and incubated for 1 h at room temperature with Alexa Fluor 488, 568, and 647 secondary antibodies (1:400, Invitrogen, USA). After washing, the specimens were counterstained with DAPI (D1306, 1:1000, Invitrogen) for 10 min. Finally, the sections were mounted and examined under a fluorescence microscope (Zeiss 880, Zeiss, Oberkochen, Germany and Leica SP5, Leica, Wetzlar, Germany). The DAPI positive percentage was determined using the ImageJ software (https://imagej.nih.gov/ij/). For statistical analysis, only one disc from each rat was analyzed (n = 4 per group).

### Antibodies and reagents

The antibodies and reagents used in this experiment are listed in Table 1.

**Table 1:** List of the antibodies and reagents

Product	Catalog number	Manufacturer
**Antibody**		
Aggrecan (rabbit, polyclonal)	ab36861	Abcam (Cambridge, UK)
Tie 2 (goat, polyclonal)	AF762	R&D systems, USA
Brachyury (mouse, monoclonal)	sc-166962	Santa Cruz, USA
Collagen type II (rabbit, polyclonal)	ab34712	Abcam (Cambridge, UK)
Collagen type I (rabbit, monoclonal)	ab270993	Abcam (Cambridge, UK)
CGRP (mouse, monoclonal)	ab81887	Abcam (Cambridge, UK)
MMP-13 (rabbit, polyclonal)	ab39012	Abcam (Cambridge, UK)
IL-1β (goat, polyclonal)	AF-501-NA	Novus Biologicals, USA
TNF-α (rabbit, polyclonal)	ab6671	Abcam (Cambridge, UK)
**Staining**		
4% paraformaldehyde phosphate buffer solution	PC2031-100–00	Biosesang, South Korea
Alexa Fluor® 488 secondary antibody	A11034	Invitrogen, USA
Alexa Fluor® 488 secondary antibody	A11029	Invitrogen, USA
Alexa Fluor® 568 secondary antibody	A10042	Invitrogen, USA
Alexa Fluor® 647 secondary antibody	A21469	Invitrogen, USA
DAPI	D1306	Invitrogen, USA

*CGRP* Calcitonin-gene related peptide, *MMP-13* Matrix metalloproteinase-13, *IL-1β* Interleukin-1β, *TNF- α* Tumor necrosis factor alpha, *DAPI* Diamidino-2-phenylinodole

### Statistical analysis

All data are expressed as the mean ± standard deviation (SD), and statistical analyses were performed using SPSS software (SPSS, Inc., USA). A t-test was used for comparisons between two groups, and a single-factor analysis of variance (ANOVA) and Tukey’s HSD post-hoc test were used to compare three or more groups. *P*-values are indicated in the caption of each figure.

## Results

### Fabrication of injectable shape-memory scaffolds

**Fig. 1:**
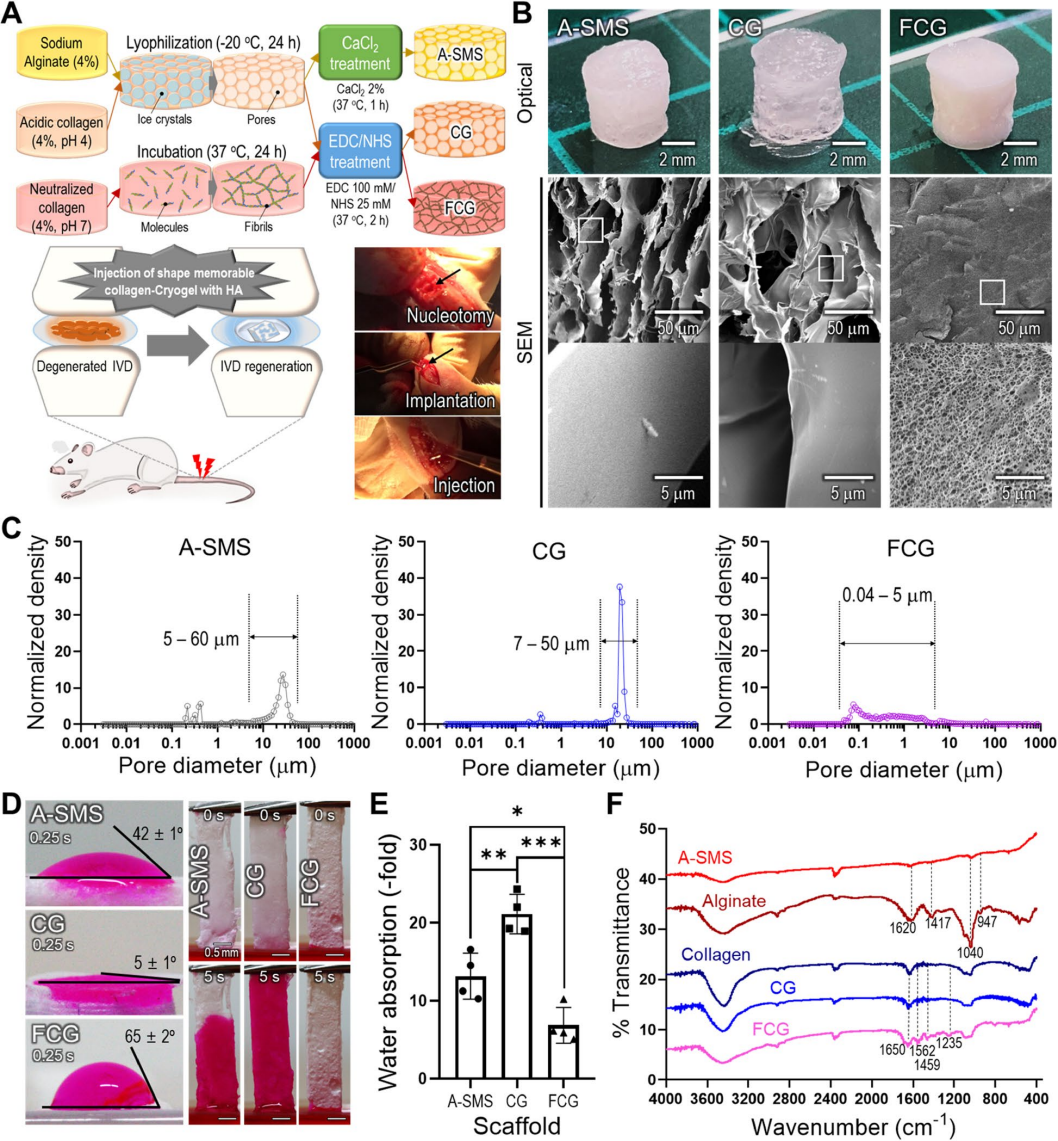
Fabrication and characterization of shape-memory structures. **A** Schematic illustrations of fabrication and in vivo procedures including nucleotomy, implantation of SMSs, and injection of HA. **B** Optical and scanning electron microscopy (SEM) images and (**C**) pore size distributions of the fabricated structures. **D** Optical images of water contact angle and absorption ability tests. **E** Quantitative water absorption (*n* = 4). **F** Fourier transform-Infrared (FT-IR) spectra of pure (sodium) alginate, collagen, and fabricated shape-memory materials. ^*^p < 0.05, ^**^p < 0.01, and.^***^p < 0.001

Generally, owing to the advantages such as minimal invasion and rapid gelation process for disc surgery, shape-memory hydrogels are of interest for application in a clinical setting [7–9]. In this study, a collagen-based structure with shape-memory was fabricated using two different procedures, as shown in Fig. 1A. In general, the pH and processing temperature greatly influences the micro/nanostructure of the collagen. To prepare the collagen shape-memory structure, collagen (4 wt%) was placed in a cylindrical mold (diameter: 5 mm and height: 5 mm) and crosslinked with the EDC/NHS solution. To obtain the CG, collagen (pH 4.0) was lyophilized at -20 °C for a day and crosslinked in the EDC/NHS solution at 37 °C for 2 h, whereas for the FCG, neutralized collagen (pH 7.8) was incubated in PBS at 37 °C for a day to obtain fibrillogenesis and then crosslinked at 37 °C for 2 h. Additionally, to obtain an A-SMS, alginate solution (4 wt%) was lyophilized at -20 °C for a day and crosslinked in CaCl_2_ solution (2 w/v%) at 37 °C for 1 h. The detailed fabrication schematic for obtaining shape-memory materials is shown in Fig. 1A. Figure 1B shows the optical and SEM images of three different shape-memory materials (A-SMS, CG, and FCG). As shown in the images, A-SMS and CG exhibited highly porous structures consisting of irregular flat sheets, whereas FCG exhibited a structure consisting of fibrillated micro/nanofibers.

Generally, the pore diameter of a shape-memory material can be a crucial factor that directly affects shape recovery through the hydration-dehydration inter-process. In particular, a larger pore size can induce a rapid rate of water filling due to capillary force; therefore, a large pore size is recommended for producing hydrogels with rapid shape recovery properties [48, 49].

Figure 1C shows the pore-size distribution in the fabricated shape-memory materials. As shown in the results, the A-SMS and CG showed pore sizes ranging from 5–60 μm, whereas that for the FCG was significantly smaller ranging from 0.04–5 μm. Based on the analysis, we expect that monolithic structures such as A-SMS and CG would have a much faster shape-recovery property compared with that of the web-like structure of FCG.

Figure 1D shows the water contact angle visualized using red rhodamine dye and optical images showing the relative water absorption ability of the structures. The CG structure with more hydrophilic and relatively larger pores showed a more efficient and faster water absorption ability (Fig. 1E). To determine whether the fabricated material was alginate or collagen, FT-IR spectroscopy was performed (Fig. 1F). Alginate showed typical absorption bands at 1620 cm^−1^ (COO- asymmetric stretching), 1417 cm^−1^ (COO- symmetric stretching), 1040 cm^−1^ (C–O–C stretching), and 947 cm^−1^ (C-O stretching) [50]. In addition, the stretching vibrations of the hydroxyl (O–H) bonds of alginate were approximately in the range of 3000–3700 cm^−1^. However, the absorption range of the O–H bonds in the spectrum of A-SMS was significantly narrower than that of pure alginate. This could be attributed to the interaction of the hydroxyl groups in alginate with calcium ions to form a chelating structure during the CaCl_2_ crosslinking process, which has been reported in other studies [51, 52]. In collagen structures, the IR spectra presented an amide-I band at 1650 cm^−1^, amide-II bands at 1562 and 1459 cm^−1^, and amide-III bands at 1235 cm^−1^ [53–58]. The results indicated that alginate and collagen-based SMSs were well fabricated through the manufacturing process without losing their chemical components.

### Shape-memory properties of fabricated alginate and collagen structures

The shape-memory property of hydrogels can be induced by entropic interaction with outwardly employed stresses, and to sustain this property, the robustly networked microporous structure should be sustained [59, 60].

**Fig. 2:**
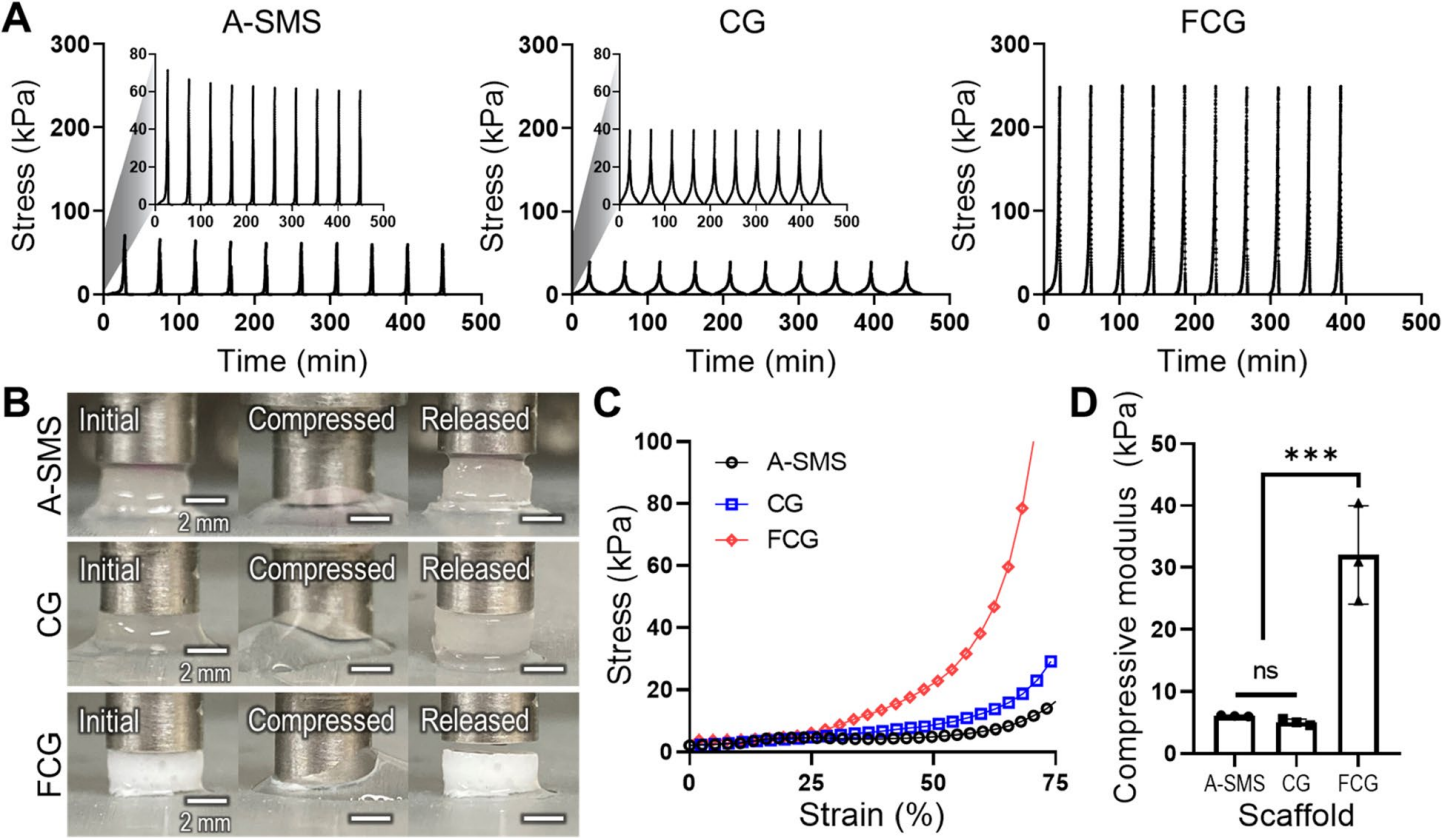
Shape-recovering properties of the fabricated structures. **A** Cyclic compressive tests (strai*n* = 90%, 10 times) and (**B**) optical images of initial, compressed (strai*n* = 90%), and released states. **C** Stress–strain curves and (**D**) compressive moduli of shape-memory structures (SMSs). Mean ± SD (*n* = 3), one-way ANOVA. ^*^p < 0.05, ^**^p < 0.01, and.^***^p < 0.001

To assess the shape-recovering property, a cylindrical shape was used for the A-SMS, CG, and FCG, and cyclic compressive stress–strain curves under 90% compressive strain were obtained by absorbing and forcing out the water from the structures (Fig. 2A). As shown by the results, all three SMSs exhibited typical shape-recovery properties. Furthermore, mechanical hysteresis, indicating the loss of energy during cyclic loading, was not assessed. Figure 2B shows optical images of the shape-recovered hydrogels. In addition, the stress–strain curves and compressive moduli of the structures were measured, as shown in Fig. 2C-D. In the results, FCG showed much greater compressive modulus compared with that of the A-SMS and CG because its web-like micro/nanoporous networks resist the externally applied forces more efficiently.

### In vitro cellular activities

**Fig. 3:**
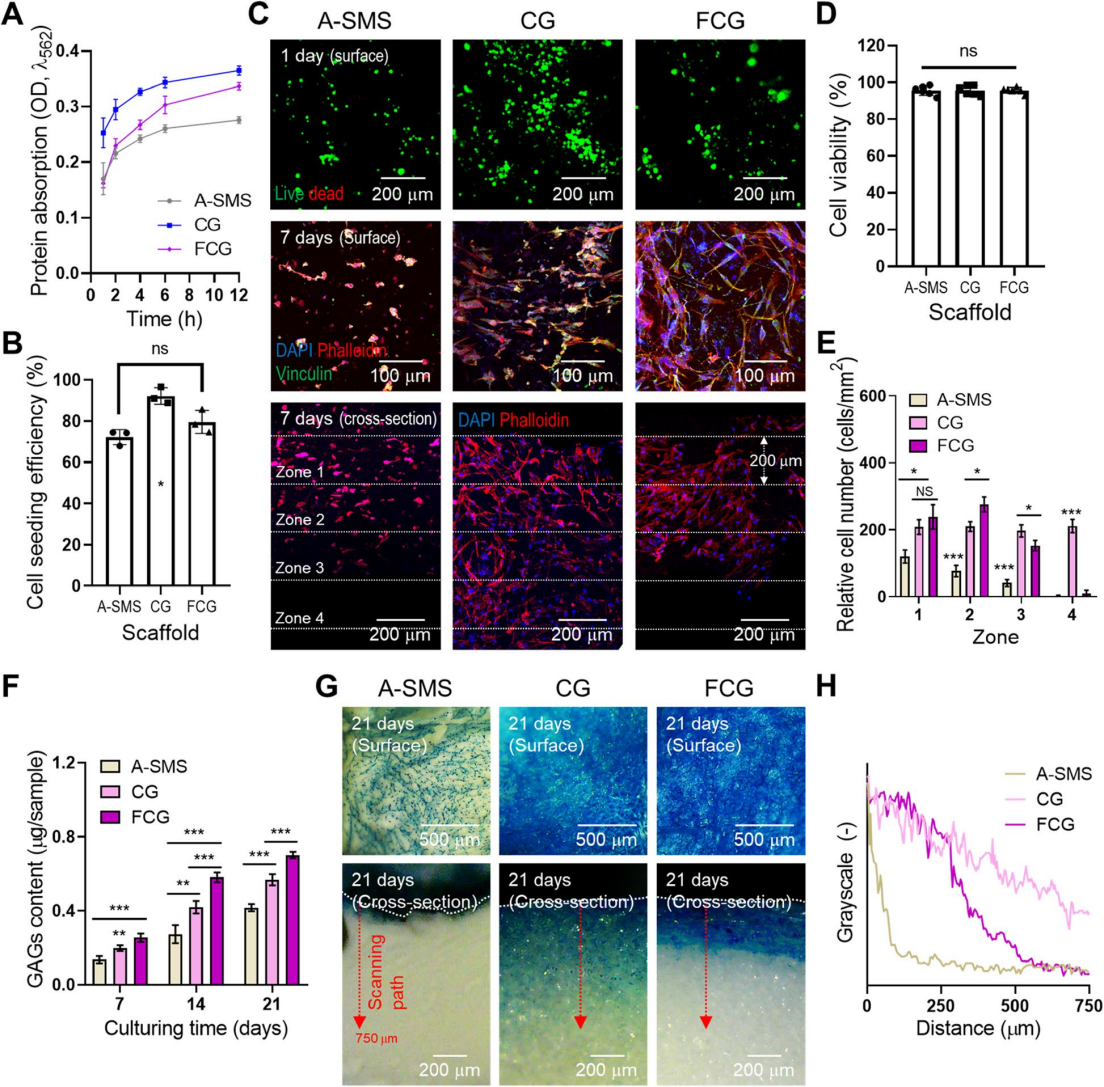
In vitro analysis. **A** Protein absorption and (**B**) cell seeding efficiency (*n* = 3). *p < 0.01, **p < 0.001, and ***p < 0.0001. **C** Fluorescent images of live/dead and diamidino-2-phenylinodole (DAPI)/phalloidin/vinculin staining. Quantitative analyses of (**D**) cell viability of cells on SMSs and (**E**) cell number per several zones. **F** Glycosaminoglycan (GAG) content (*n* = 6). ^*^p < 0.05, ^**^p < 0.01, and ^***^p < 0.001. **G** Optical images and (**H**) greyscale plots of Alcian blue staining

The protein absorption ability of tissue engineered scaffolds is highly correlated with chemical composition, pore geometry, roughness, and hydrophobicity [61]. This property can be directly attributed to the initial cell attachment and activation capability of several multifunctional glycoproteins that attract seeded cells [62]. The absorption properties of the structures were evaluated for up to 12 h (Fig. 3A). A much higher absorption ability was observed for the CG structure, whereas that of A-SMS and FCG was significantly lower. This was due to the synergistic effect of the biochemical components of collagen and the much higher porosity of the CG structure. The result correlated well with the initial cell seeding efficiency, which was measured on day 1 (Fig. 3B), indicating that the CG structure showed significantly higher cell seeding efficiency compared with that of the other structures.

The fundamental cytocompatibility of hASCs cultured on the structures is shown in Fig. 3C. It was evaluated based on live (green)/dead (red) staining on day 1 and DAPI (blue)/phalloidin (red)/vinculin (green) staining on day 7. The fluorescence images indicated a healthy dominance of live cells in all structures (Fig. 3D). However, the staining of F-actin and vinculin that is key cell adhesion protein showed that the cells cultured on CG and FCG exhibited a stretched spindle-shaped micromorphological structure, whereas those cultured on A-SMS had round-shaped morphologies. Furthermore, to observe cell proliferation and extent of migration with regard to the thickness of the structures and direction of penetration, we analyzed the cross-sectional view of the DAPI/phalloidin stained structures on day 7. As shown in Fig. 3C, we divided the cultured structures into four depth zones (distance of each zone: 200 μm). Interestingly, the CG structure showed relatively deeper cell migration and proliferation compared with that in FCG and a more activated F-actin cytoskeleton compared with that of the A-SMS structure. For the FCG structure, the proliferating cells showed less migration into the scaffold thickness compared with that in the CG structure (Fig. 3E). This could be attributed to the much larger pore size in the CG structure than in the FCG structure.

To examine the biological responses of the structures to chondrogenic differentiation of the cultured hASCs, Alcian blue staining was performed to investigate in vitro cellular activities based on the GAG content. The GAG content of the structures was measured on days 7, 14, and 21 (Fig. 3F). As shown in the results, FCG showed the highest GAG content owing to its highly cell-favorable micro/nanofibrous structure. We performed Alcian blue staining to observe the surface and cross-sectional distribution of GAG generated by hASCs in the structures (Fig. 3G). The GAG content was significantly more homogeneous in the CG structure than in the FCG and A-SMSs in the cross-sectional view, although FCG showed the highest GAG production on the surface of the structure (Fig. 3H).

### In vivo study in rat tail nucleotomy model

**Fig. 4:**
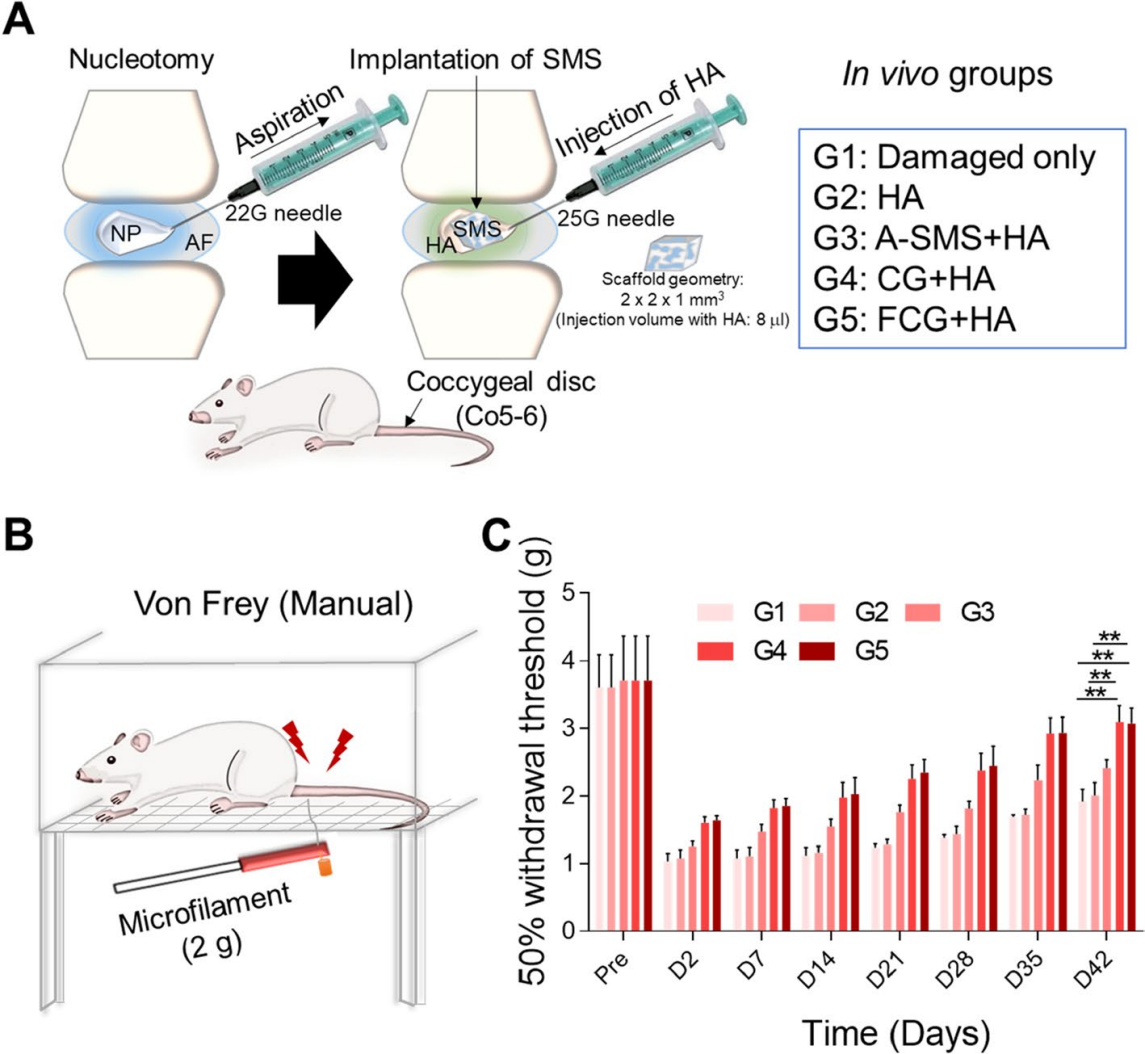
In vivo study. **A** Schematic illustration of nucleotomy performed on eight-week-old female Sprague Dawley rats and treatment of in vivo groups. **B** Schematic image of von Frey test by using 2 g microfilament. **C** Characterization of a pain model induced by intervertebral disc (IVD) injury. The von Frey test indicated mechanical allodynia in rat model. Mean ± SD, (*n* = 6), one-way repeated measures of ANOVA

Along with the in vitro data, the evaluation of SMSs (A-SMS, CG, and FCG) was performed in vivo using a nucleotomy model in a rat tail disc. The in vivo procedures including nucleotomy, implantation of SMSs, and injection of HA were performed in the coccygeal disc (Co5-6) of 40 Sprague Dawley rats, divided into five groups: damaged only (G1), treated with hyaluronic acid (HA) (G2), A-SMS with HA (G3), CG with HA (G4), and FCG with HA (G5), as shown in Fig. 4A. In addition, we performed the von Frey test in all groups of animals (*n* = 6) 2 days before nucleotomy (pre), then on days 2, 7, 14, 21, 28, 35, and 42 to check for mechanical allodynia in rats (Fig. 4B). Our findings showed that the 50% withdrawal threshold significantly increased from day 2 to day 42 after damage and treatment. G4 and G5 showed a significantly higher 50% withdrawal threshold than the other groups (G1, G2, and G3), which signifies that treatment with collagen structures (CG and FCG) alleviated mechanical allodynia in the rat tail (Fig. 4C).

**Fig. 5:**
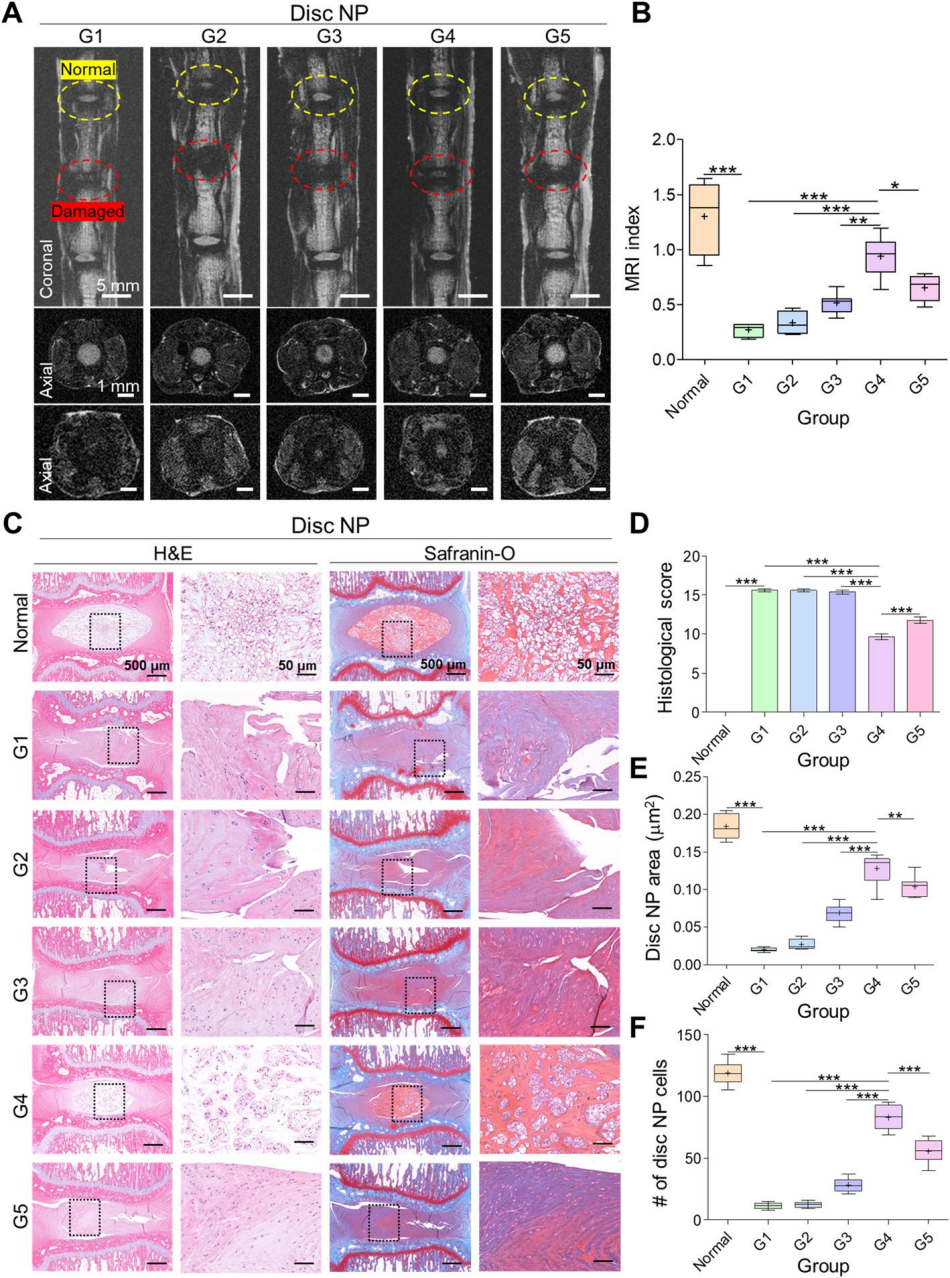
MRI and histological analyses. **A** Representative T2-weighted magnetic resonance imaging (MRI) of the coccygeal discs: normal and damaged (sagittal view, yellow circle). **B** MRI index for discs of each group. Scale bars: coronal 5 mm, axial 1 mm. **C** Representative images of hematoxylin and eosin (H&E) and Safranin O staining. H&E disc nucleus pulpous (NP) area and cell number. **D** Histological score analysis from Safranin O staining. **E** Quantitative analysis of H&E revealed the highest preservation of disc structure in G4. **F** The highest disc cell number is revealed in normal disc followed by G4. Mean ± SD (*n* = 8), one-way ANOVA, scale bars: 500 μm, 50 μm

For further experiments, the animals were euthanized after six weeks. 9.4 T MRI of the disc was performed, and the T2-weighted coronal and axial plane images of each group are shown in Fig. 5A. The normal healthy disc from all groups showed a bright signal intensity in the NP region, indicated by yellow circles, whereas the damaged and treated groups showed dark signal intensity in the NP region, indicated by red circles (Fig. 5A). The area of the NP and ROI ratio of the damaged discs were compared with the value of the normal disc to evaluate the MRI index. No significant difference was observed between G1 and G3, whereas G4 showed the brightest intensity among the groups and exhibited the closest values to the normal disc, as shown in Fig. 5B. This indicates that combined treatment with CG and HA would preserved more of the hydration of the disc and enhance disc regeneration.

After MRI analysis, the isolated disc was assessed using histological analysis to determine the tissue structure and morphology. We performed Hematoxylin and Eosin (H&E) and Safranin-O staining (Fig. 5C), and calculated histological scores (Fig. 5D), the disc NP area (Fig. 5E), and cell number (Fig. 5F) to reveal PG distribution in disc NP. After surgery, the damaged disc (untreated) appeared as annular ruptured clustered NP cells, whereas the normal healthy disc comprised well-organized AF tissue and NP cellularity without cell clusters. H&E and Safranin-O staining results showed significantly decreased intensity and significantly increased histological grade in the G1 group, indicating decreased PG content and less preservation of the disc structure. Consistent with the MRI data, not many differences were found between G1, G2, and G3. However, G4 showed a significantly decreased histological grade compared with that of the other groups, including G5. Furthermore, H&E staining showed delayed loss of NP-positive area and increased disc NP cell number in G4 due to reduced cell loss in NP and better maintenance of the disc structure and PG matrix than that in the other groups (Fig. 5C-F).

**Fig. 6:**
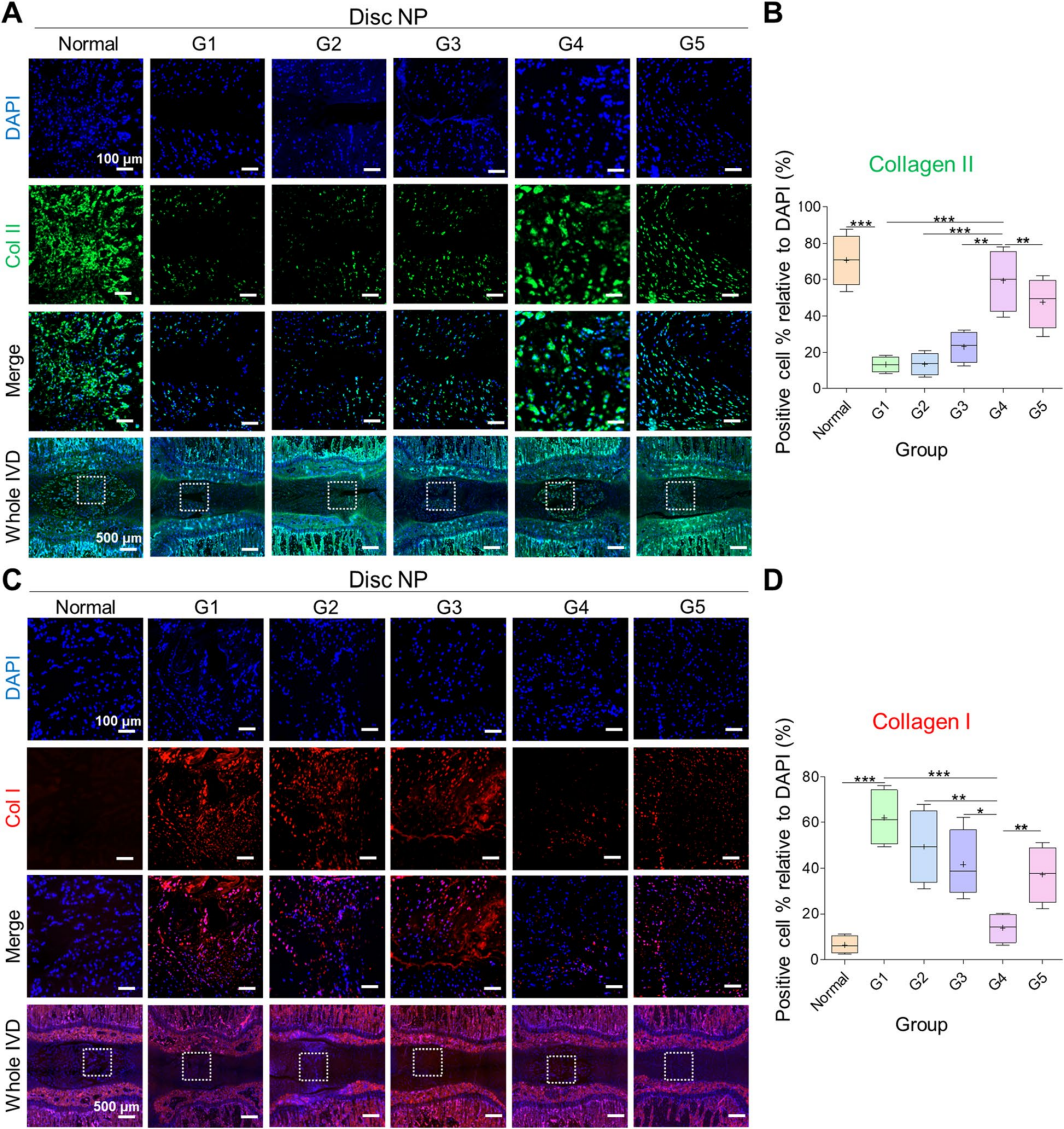
Immunofluorescent analyses of collagen type II and I. **A**, **C** Representative images and **B**, **D** quantitative analyses. The highest and lowest percentage of cell number was observed in normal disc followed by G4 treated disc, whereas the lowest and highest was seen in G1 in collagen II and I, respectively. Mean ± SD (*n* = 4), one-way ANOVA. Scale bar: 100 μm, 500 μm

Next, we performed immunofluorescence to evaluate matrix protein restoration after treatment for six weeks. Our results showed that the percentage of positive cells relative to that of DAPI of collagen type II was significantly higher in the normal disc than in the damaged disc. Similarly, G4 showed a significantly higher percentage of collagen type II cells relative to that of DAPI than in the other groups, including G5 (**p < 0.01 G4 vs. G5) (Fig. 6A-B). However, type I collagen positive cell percentage relative to that of DAPI was significantly higher after damage than in normal discs (***p < 0.001 damaged vs. normal). Moreover, G4 showed a significantly lower percentage of collagen type I cells relative to that of DAPI than the other groups, including G5 (**p < 0.01 G4 vs. G5) (Fig. 6C-D). Overall, G4 showed restoration of the matrix protein and better maintenance of the disc NP structure, showing an increased degree of collagen type II and gradual loss of collagen type I after 6 weeks of treatment.

**Fig. 7:**
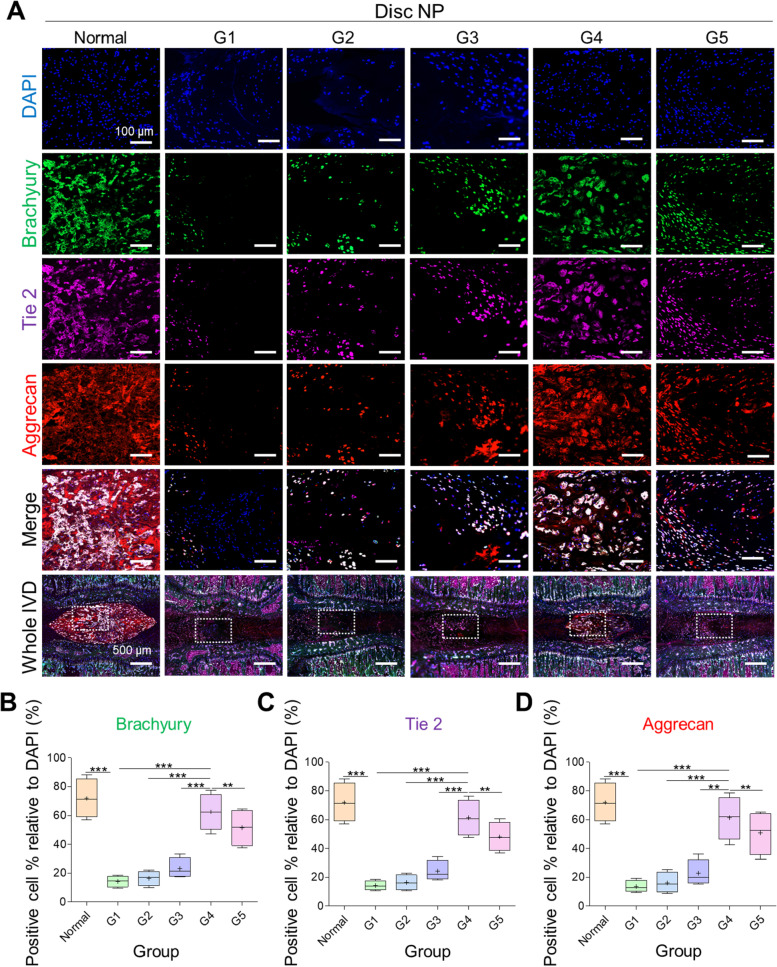
Immunofluorescent analyses of brachyury, Tie-2, and aggrecan. **A** Representative images and **B**-**D** quantitative analyses. The highest percentage of cell number was observed in normal disc followed by G4 treated disc, whereas the lowest was seen in G1. Mean ± SD (*n* = 4), one-way ANOVA. Scale bar: 100 μm, 500 μm

We performed immunofluorescence for detecting brachyury, which plays an important role in notochord cell formation, Tie-2, which aids cell proliferation and migration, and aggrecan, to evaluate the restoration of ECM and disc NP cells in which it is mostly contained. Our results showed a significantly lower percentage of brachyury, Tie-2, and aggrecan in damaged discs than in normal discs (***p < 0.0001 damaged vs. normal). In contrast, G4 showed significantly higher brachyury-, Tie-2-, and aggrecan-positive cell percentages than that of the other groups, including G5 (**p < 0.01 G4 vs. G5) (Fig. 7A-D), indicating that G4 showed upregulated disc matrix restoration.

**Fig. 8:**
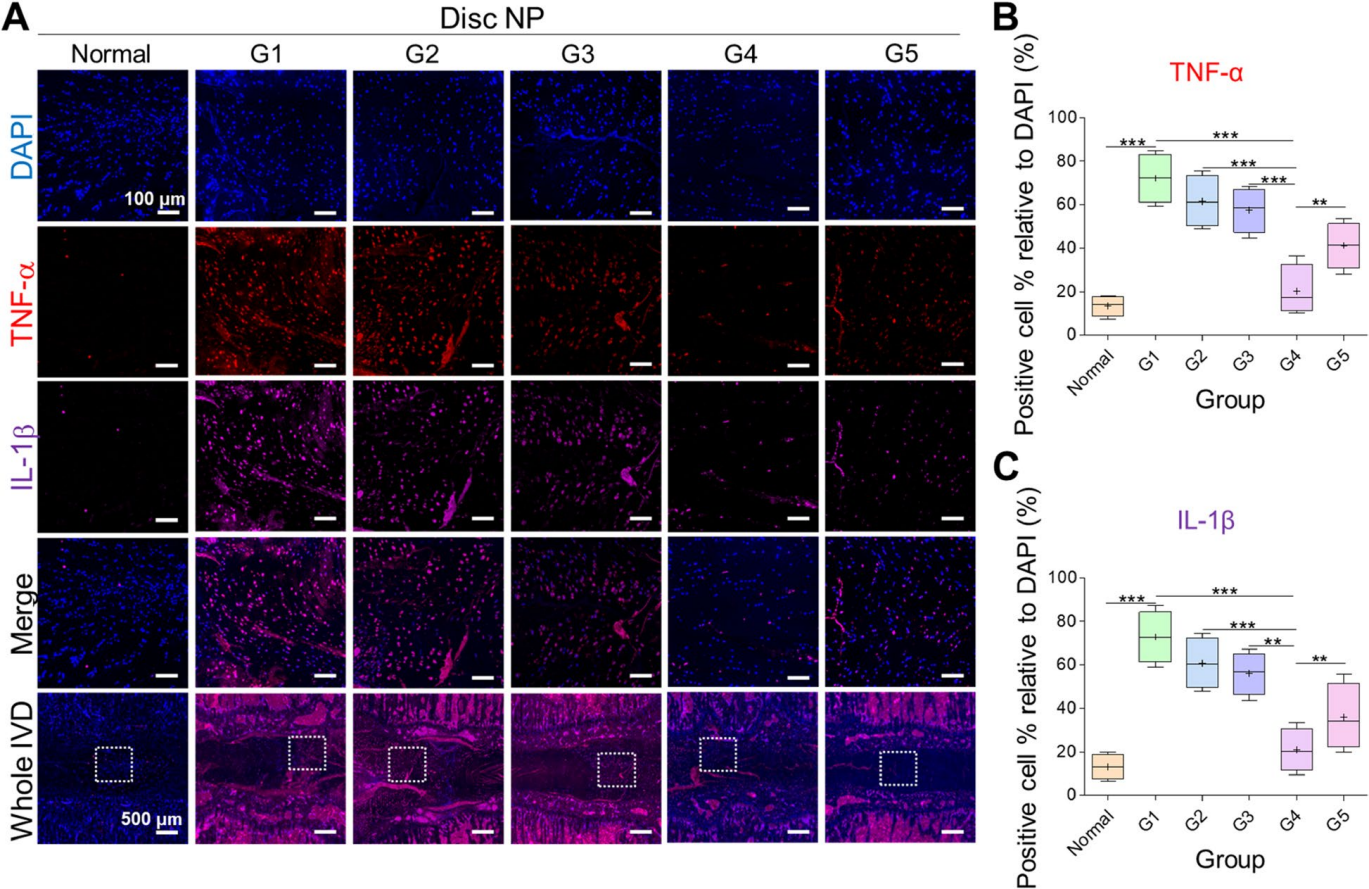
Inflammatory responses. **A** Representative images and (**B**, **C**) quantitative analyses of tumor necrosis factor alpha (TNF-α) and interleukin-1β (IL-1β). The lowest percentage of cell number was observed in normal disc followed by G4 treated disc, whereas the highest was seen in G1 damaged disc. Mean ± SD (*n* = 4), one-way ANOVA. Scale bar: 100 μm, 500 μm

Furthermore, we evaluated the inflammatory response in each group six weeks after surgery. We performed immunofluorescence for the proinflammatory cytokines tumor necrosis factor alpha (TNF-α) and interleukin-1β (IL-1β). The results showed significantly higher TNF-α and IL-1β cell percentage in the damaged discs than in the normal discs (***p < 0.001 damaged vs. normal). However, the percentages of TNF-α and interleukin-1β positive cells were significantly lower in G4 than in the other groups, including G5 (**p < 0.01 G4 vs. G5) (Fig. 8A-C). The results showed that inflammatory cytokines were downregulated after combined treatment with CG and HA.

**Fig. 9:**
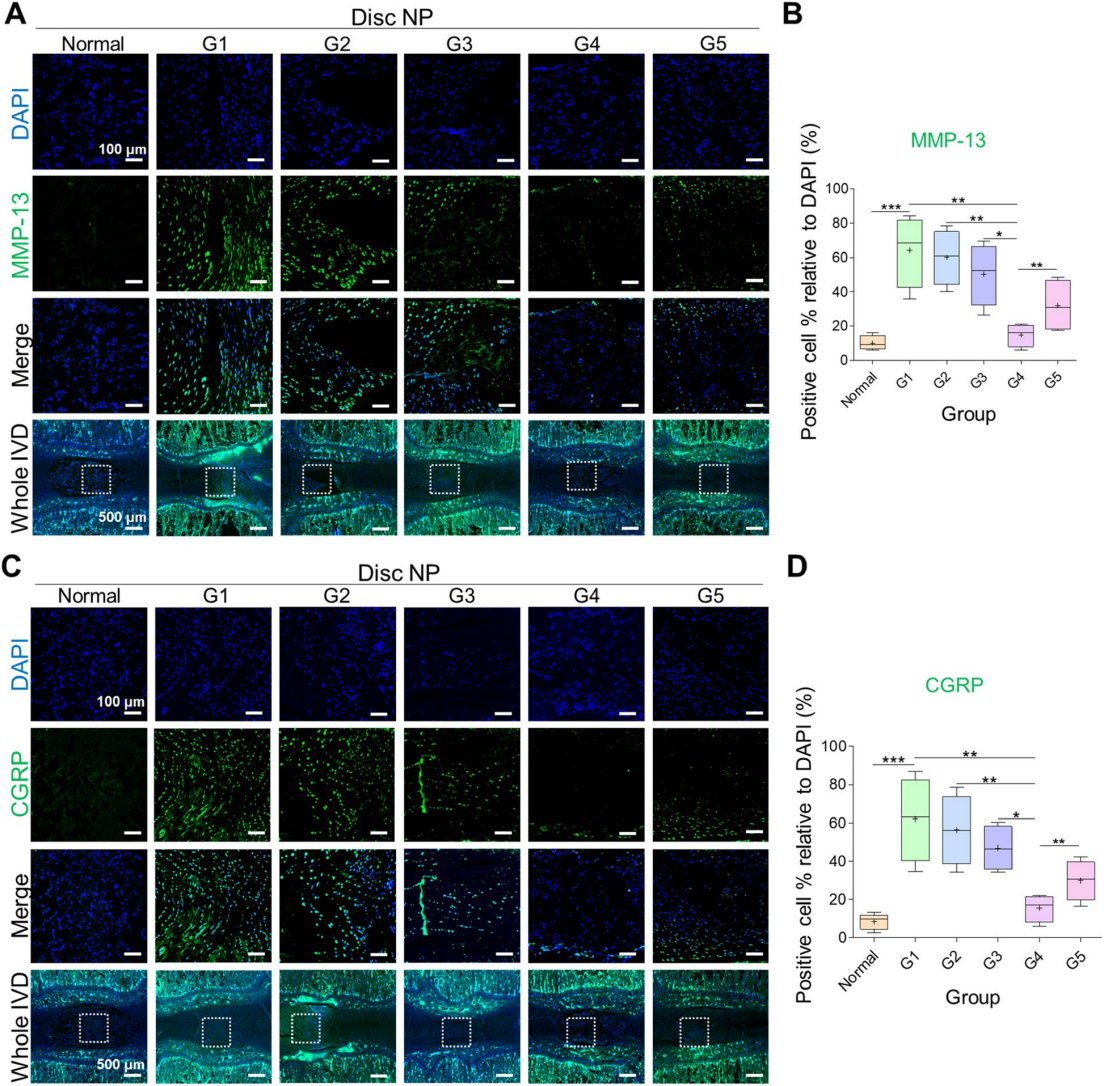
Disc degeneration and damage-induced pain analyses. **A**, **C** Representative images and **B**, **D** quantitative analyses of matrix metalloproteinase-13 (MMP-13) and neuropeptide calcitonin gene receptor protein (CGRP). The lowest percentage of cell number was observed in normal disc followed by G4 treated disc, whereas the highest was seen in G1. Mean ± SD (*n* = 4), one-way ANOVA. Scale bar: 100 μm, 500 μm

In addition, we performed immunofluorescence staining for matrix metalloproteinase enzyme (MMP-13), a key enzyme involved in the degradation of collagenous ECM in cartilage tissue during disc degeneration. We detected a significantly increased percentage of cartilaginous disc destruction in the damaged disc tissue compared with that in the normal (healthy) disc tissue (***p < 0.0001 damaged vs. normal). Similarly, G4 showed a significantly decreased percentage of MMP-13 cells compared with that of the other groups, including G5 (**p < 0.01 G4 vs. G5) (Fig. 9A-B). These results confirmed that CG combined with HA treatment inhibited the destruction of the collagenous cartilage of the disc tissue by maintaining the disc matrix.

Additionally, we performed immunofluorescence staining of the sensory neuropeptide calcitonin gene receptor protein (CGRP) to evaluate the damage-induced pain phenotype. We observed a significantly increased cell percentage pertaining to CGRP in the damaged (untreated) disc compared with that in the normal (healthy) disc (***p < 0.001 damaged vs. normal). However, the discs in G4 showed a significantly decreased cell percentage corresponding to CGRP compared with that of the discs in the other groups, including G5 (**p < 0.01 G4 vs. G5) (Fig. 9C-D). The results showed that CG combined with HA treatment reduced the damage-induced pain phenotype.

## Discussion

Chronic LBP is the most common cause of IVD degeneration and a socioeconomic burden worldwide [63, 64]. As degeneration progresses, matrix protein degradation occurs, which causes painful inflammation. The alleviation of pain is the primary step for functional recovery in patients with damaged discs.

As the breakdown of collagen induces IVD degeneration, using collagen hydrogel is a promising approach for successfully regenerating AF and NP tissues [14]. Previously, we showed that porous collagen structures crosslinked with EDC/NHS solution are suitable biomedical scaffolds for the regeneration of various tissues [33]. In particular, collagen is versatile, and the micro/nanostructures can be controlled when processed with various fabrication procedures using different pH and processing temperature, as shown in Fig. 1A. The resulting porous collagen structures are mechanically stable, and they present a typical shape-memory property through the hydration-dehydration process (Fig. 2). The biochemical and shape-memorability properties of the fabricated collagen scaffold are extremely alluring for applications that induce the regeneration of damaged IVD or in minimally invasive surgery. Additionally, the collagen scaffold can easily encase a defective region in a variety of sizes and complex geometries.

To evaluate the feasibility of the shape-memory collagen scaffold for in vitro biological activities, we prepared three different types of shape-memory hydrogels: alginate (A-SMS) and two porous collagen structures CG and FCG, as shown in Fig. 1. The conventional alginate-based shape-memory structure (A-SMS) and CG structure exhibited similar physical properties in terms of water absorption, compressive properties, and shape-memorability (Figs. 1 and 2). In addition, collagen-based SMSs (CG and FCG) showed significantly higher cellular activities and regulated cellular behavior in terms of cell shape and morphology compared with that of the A-SMS. In particular, the hASCs cultured in the A-SMS demonstrated a rounded morphology, whereas in collagen-based SMSs, the cells adhered well and presented well-stretched fibroblastic-like phenotypes that are typical to the morphology of AF cells. However, the chondrogenic activities of the collagen-based SMSs were significantly different. In the CG structure, the observed PG production was homogeneous across both the surface and across the depth/thickness of the structure, whereas in the fibrous FCG scaffold, the PG were distributed only on the surface. Although the fibrillated collagen of the FCG structure enables more efficient cell adhesion and proliferation, the inconvenient pore geometry (i.e., low pore size distribution) hinders effective cell infiltration or migration into the structure. According to the biophysical and in vitro results, we hypothesize that the porous CG efficiently and homogeneously induces chondrogenic activities without loss of shape-memory properties.

HA, a hydrophilic molecule and an abundant polymer in the ECM, is commonly used as an anti-inflammatory and analgesic biomolecule, mostly in cartilage tissue repair [43, 65, 66]. Over the past few decades, HA alone or in combination with stem cells has been clinically tested in an IVDD model [23]. To accelerate the regeneration of the damaged IVD and to avoid the risk of scaffold from stuck in needle while injecting together with HA, we first implanted the regenerative scaffolds and then immediately injected the HA solution (1 w/v% in PBS) for the absorption of scaffold to regain its original shape in the damaged IVD during the in vivo experiments.

In in vivo studies, we assessed pain behavior in rats 2 day before surgery (nucleotomy followed by implantation) and for at least 6 weeks after implantation. The rats were then euthanized, and the tissues were collected for MRI, histological, and immunofluorescence analysis. The major observations of this study with regard to the effects exerted by the hydrogels are as follows: (1) alleviation of mechanical allodynia in a rat tail nucleotomy model [67]; (2) restoration of the disc anatomy and water content of IVDs; (3) preservation of the PG content and disc structure; (4) restoration of the matrix protein and maintenance of the disc NP structure; (5) preservation of the disc NP cells; (6) downregulation of the inflammatory cytokines; (7) reduction in neuropathic pain; and (8) inhibition of the MMP enzymes.

First, we performed the von Frey test to check for mechanical allodynia in the rats. The results showed that in G4, CG combined with HA showed a significantly higher 50% withdrawal threshold than that in G1, indicating that treatment with this structure can alleviated mechanical allodynia in the rat tail. A similar result was reported when HA hydrogel was used in a puncture model of rat implantation; it lowered the mechanical allodynia in rats after injury [43].

A high water content in the IVD indicates greater elasticity under externally applied load-bearing capacity, and loss of water implies the progression of disc degeneration [68]. The T2-weighted MRI after six weeks of treatment showed bright signal intensity in G4 treated discs compared with discs treated with HA alone (G2), A-SMS combined with HA (G3), and FCG with HA (G5), indicating recovery after treatment and more water retention in the disc. In a healthy IVD, a distinct border is discerned between the annulus fibrosus (AF) and nucleus pulposus (NP); however, it gradually disappears in a degenerated disc [69, 70]. In our study, the results of the histological staining showed a more distinct border between NP and AF with increased PG content and better-preserved disc structure in the G4 implantation disc.

ECM is a major component of the NP and contains PGs, such as aggrecan and collagens (type I mostly found in AF and type II found in NP). Type II collagen is the most sensitive collagen in the NP, and it forms an irregular structure that maintains the disc PGs [3, 71]. During degeneration, type II collagen decreases, and type I collagen increases with a simultaneous loss of biomechanical function in NP. Immunofluorescence results in G4 showed increased level of collagen type II and gradual loss of collagen type I after six-weeks of implantation. Similarly, immunofluorescence results for brachyury, which is important for notochordal cell formation, tie-2 for cell proliferation and migration, and aggrecan, an important ECM component, showed a high percentage of disc NP cells in the G4 treated disc post damage. Furthermore, this study is comparable to a previous study on low adhesive collagen scaffold injected disc, which indicated that NP cells positive for brachyury and tie-2 contribute to disc repair by expressing aggrecan and collagen type II post damage [16].

Furthermore, we performed immunofluorescence analysis for the pro-inflammatory cytokines TNF-α and IL-1β, which are expressed after disc damage. The results revealed that pro-inflammatory cytokines were downregulated after treatment in G4, which enhanced the regeneration of the IVD. A previous study demonstrated that HA is involved in an anti-inflammatory mechanism that helps reduce pain [43]. Moreover, the neurogenic factors that are released after disc damage can sensitize nociceptors and initiate nerve transmission to the spinal cord [72], and these neurogenic factors (neuropeptides and CGRP) can trigger discogenic back pain [73]. Our results showed that the high percentage of cells in the damaged and treated disc in G4 reduced pain. In contrast. a previous study reported that the implantation of collagen/HA hydrogel inhibited the sensory neuropeptide in a rat model of punctured discs [43]. Our results confirmed that implantation of CG and HA inhibited the destruction of the collagenous cartilage of the disc tissue by maintaining the disc matrix. Although FCG group (G5) was superior to A-SMS group (G3) in disc repair and pain reduction, CG group (G4) was superior to FCG group (G5) in disc repair and pain reduction.

This study has some limitations, such as the fact that rodents have notochordal cells in their disc for a lifetime and have a lower risk for disc degenerative diseases compared with humans. In addition, the rat tail disc is structurally different from the lumbar discs of rats and humans [74]. Moreover, the surgical method for inducing disc damage in the rat tail does not mimic the process that leads to degenerated and herniated discs clinically observed in humans [75]. Therefore, preclinical tests need to be performed in larger animals. Lastly, only NP region of IVD was targeted using A-SMS, CG, and FCG with HA, regardless of the difference of shape-memory behaviors between the structures. Even though FCG group (G5) has shown relatively disappointing results compared to CG group (G4) in this study, however, it showed alleviated mechanical allodynia and enhanced disc regeneration compared to A-SMS group (G3) and can be applied to the fibrous AF tissue repair for the whole IVD regeneration in the future. Thus, in this study, we have developed collagen-based SMSs combined with HA to enhance IVD regeneration and reduced inflammation and pain.

## Conclusions

This study demonstrated that collagen-based SMSs combined with HA possess higher efficacy for the treatment of disc degeneration. The G4 group demonstrated alleviated mechanical allodynia in a rat tail nucleotomy model, preserved water content in the disc, reduced histological damage by maintaining PGs, and restored matrix proteins by preserving NP cells. Our results suggest that the combination of HA and collagen-based SMS, in particular CG, can induce IVD repair more efficiently to promote disc regeneration and reducing pain.

## Data Availability

The data supporting the conclusions of this study are available with the corresponding author upon reasonable request.

## Abbreviations

3DW: Triple-distilled water
AF: Annulus fibrosus
ANOVA: Analysis of variance
A-SMS: Alginate shape-memory structure
BCA: Bicinchoninic acid
BSA: Bovine serum albumin
CaCl2: Calcium chloride
CG: Collagen-cryogel
CGRP: Calcitonin-gene related peptide
DAPI: Diamidino-2-phenylinodole
DMEM: Dulbecco’s modified Eagle’s medium
ECM: Extracellular matrix
EDC: 1-Ethyl-3-(3-dimethylaminopropyl) carbodiimide hydrochloride
EDTA: Ethylenediaminetetraacetic acid
FBS: Fetal bovine serum
FCG: Fibrous collagen-gel
FT-IR: Fourier-transform infrared
GAG: Glycosaminoglycan
H&E: Hematoxylin and eosin
HA: Hyaluronic acid
hASC: Human adipose-derived stem cell
IL-1β: Interleukin-1β
IVD: Intervertebral disc
IVDD: Intervertebral disc degeneration
LBP: Low back pain
MMP: Matrix metalloproteinase
MRI: Magnetic resonance imaging
MSC: Mesenchymal stem cell
NBF: Neutral buffered formalin
NHS: N-hydroxy-succinimide
NP: Nucleus pulpous
PBS: Phosphate-buffered saline
PFA: Paraformaldehyde
PG: Proteoglycan
PLA: Polylactic acid
PS: Penicillin/streptomycin
ROI: Region of interest
SD: Standard deviation
SEM: Scanning electron microscopy
SMS: Shape-memory structure
TE: Time to echo
TGF-β1: Transforming growth factor-β1
TNF-α: Tumor necrosis factor alpha
TR: Time to repetition
UTM: Universal testing machine
